# Supplementary material for: Polyketide synthase gene clusters in the ericoid mycorrhizal fungus Oidiodendron maius: genome-wide prediction, expression profiling, and functional characterization of knockout mutants
Source: Front Plant Sci. 2026 Sep 16;17:1924430. doi: 10.3389/fpls.2026.1924430 (PMC13623903; doi:10.3389/fpls.2026.1924430)
Supplement: Supplementary file 1 [file SupplementaryFile1.zip › Supplementary Materials/Supplementary Folder 1.zip!/Oidma1_AssemblyScaffolds_Repeatmasked/knownclusterblast/cluster56/jgi.p_Oidma1_185003_mibig_hits.html]

| MIBiG Protein | Description | MIBiG Cluster | MiBiG Product | % ID | % Coverage | BLAST Score | E-value |
| --- | --- | --- | --- | --- | --- | --- | --- |
| CRG85577.1 | hypothetical\_protein | BGC0001402 | Cyclochlorotine\_biosynthetic\_gene\_cluster | 32 | 83.1141868512 | 524 | 4.2e-148 |
| AMM63163.1 | AniL | BGC0001371 | Echinocandin\_B\_biosynthetic\_gene\_cluster | 28 | 63.6678200692 | 305 | 3.1e-82 |
| AFT91383.1 | EcdL | BGC0000338 | Echinocandin\_B\_biosynthetic\_gene\_cluster | 27 | 65.1903114187 | 293 | 1.2e-78 |
| OAQ83754.1 | ABC\_multidrug\_transporter | BGC0001358 | Leucinostatins\_biosynthetic\_gene\_cluster | 27 | 64.9826989619 | 289 | 2.3e-77 |
| EPE34342.1 | P-loop\_containing\_nucleoside\_triphosphate\_hydrolase | BGC0001035 | Pneumocandin\_biosynthetic\_gene\_cluster | 27 | 66.4359861592 | 272 | 3.8e-72 |
| AAN74822.1 | Fum19p | BGC0000062 | Fumonisin\_biosynthetic\_gene\_cluster | 24 | 83.5294117647 | 255 | 2.8e-67 |
| ACB12564.1 | Fum19 | BGC0000063 | Fumonisin\_biosynthetic\_gene\_cluster | 24 | 82.9757785467 | 252 | 4e-66 |
| EFY94505.1 | multidrug\_resistance\_protein | BGC0000337 | Destruxins\_biosynthetic\_gene\_cluster | 25 | 70.4498269896 | 192 | 2.9e-48 |
| CAC20925.1 | PimB\_protein | BGC0000125 | Pimaricin\_biosynthetic\_gene\_cluster | 29 | 38.892733564 | 155 | 3.9e-37 |
| CAG38700.1 | putative\_ABC-transporter,\_ATP-binding\_protein | BGC0000708 | Lividomycin\_biosynthetic\_gene\_cluster | 27 | 37.7162629758 | 152 | 3.3e-36 |
| ADX66465.1 | ScnB | BGC0000108 | Natamycin\_biosynthetic\_gene\_cluster | 28 | 38.9619377163 | 152 | 4.3e-36 |
| AAK73498.1 | AmphG | BGC0000015 | Amphotericin\_biosynthetic\_gene\_cluster | 27 | 38.4775086505 | 141 | 5.9e-33 |
| ANP43732.1 | ThsT | BGC0001391 | Thusin\_biosynthetic\_gene\_cluster | 25 | 40.0692041522 | 140 | 1.3e-32 |
| AGM16416.1 | paenibacterin\_ABC-transporter\_E | BGC0000400 | Paenibacterin\_biosynthetic\_gene\_cluster | 27 | 34.3944636678 | 140 | 1.7e-32 |
| ADX66464.1 | ScnA | BGC0000108 | Natamycin\_biosynthetic\_gene\_cluster | 30 | 23.321799308 | 140 | 1.7e-32 |
| AAF71763.1 | NysG | BGC0000115 | Nystatin\_biosynthetic\_gene\_cluster | 26 | 37.0934256055 | 139 | 2.2e-32 |
| AMR44284.1 | ABC\_transporter | BGC0001398 | Phomopsins\_biosynthetic\_gene\_cluster | 25 | 58.7543252595 | 139 | 2.2e-32 |
| AET14838.1 | multidrug\_resistance\_protein | BGC0001274\_c2 | Non-acetylated\_open-chain\_sophorolipid\_/\_diacetylated\_lactonic | 30 | 26.0207612457 | 136 | 2.5e-31 |
| AIS24845.1 | Dst21 | BGC0001147 | Distamycin\_biosynthetic\_gene\_cluster | 26 | 35.7785467128 | 136 | 3.2e-31 |
| ANQ31705.1 | ABC\_transporter | BGC0001395 | Teichomycin\_biosynthetic\_gene\_cluster | 27 | 36.0553633218 | 135 | 5.5e-31 |
| CAF31837.1 | putative\_hygromycin\_B\_exporter | BGC0000699 | Hygromycin\_B\_biosynthetic\_gene\_cluster | 26 | 33.8408304498 | 134 | 7.2e-31 |
| BAI23327.1 | putative\_ABC-transporter | BGC0000949 | A-500359s\_biosynthetic\_gene\_cluster | 24 | 39.2387543253 | 133 | 2.1e-30 |
| CAC20924.1 | PimA\_protein | BGC0000125 | Pimaricin\_biosynthetic\_gene\_cluster | 29 | 22.76816609 | 133 | 2.1e-30 |
| CAI47637.1 | putative\_ABC-type\_aminoglycoside\_exporter | BGC0000692 | Apramycin\_biosynthetic\_gene\_cluster | 25 | 37.7162629758 | 132 | 3.6e-30 |
| ABV83218.1 | CppG | BGC0000116 | Nystatin-like\_Pseudonocardia\_polyene\_biosynthetic\_gene\_cluster | 29 | 37.8546712803 | 132 | 3.6e-30 |
| CAG34716.1 | putative\_ABC\_transporter | BGC0000713 | Ribostamycin\_biosynthetic\_gene\_cluster | 25 | 39.1003460208 | 131 | 6.1e-30 |
| AAZ76601.1 | BhtT | BGC0000497 | BhtA1\_/\_BhtA2\_biosynthetic\_gene\_cluster | 27 | 32.3183391003 | 131 | 6.1e-30 |
| BAB04170.1 | lantibiotic\_mersacidin\_transporter\_system | BGC0000517 | Haloduracin\_alpha\_/\_haloduracin\_beta\_biosynthetic\_gene\_cluster | 24 | 36.8166089965 | 131 | 6.1e-30 |
| BAD72775.1 |  | BGC0000552 | Smb\_A\_/\_Smb\_B\_biosynthetic\_gene\_cluster | 27 | 32.3183391003 | 131 | 7.9e-30 |
| AAK81823.1 | ABC\_transporter | BGC0000326 | Complestatin\_biosynthetic\_gene\_cluster | 25 | 40.553633218 | 131 | 1e-29 |
| XP\_011392729.1 | ABC\_transporter | BGC0001281 | Ustilagic\_acid\_biosynthetic\_gene\_cluster | 30 | 24.4982698962 | 129 | 3e-29 |
| AAC44070.1 | unknown | BGC0000797 | Sphingan\_polysaccharide\_biosynthetic\_gene\_cluster | 26 | 37.3702422145 | 129 | 3e-29 |
| EEM14530.1 | hypothetical\_protein | BGC0001210 | Pseudomycoicidin\_biosynthetic\_gene\_cluster | 25 | 36.3321799308 | 129 | 3e-29 |
| AFJ14797.1 | PlpH | BGC0000403 | Pelgipeptin\_biosynthetic\_gene\_cluster | 30 | 20.3460207612 | 128 | 5.1e-29 |
| AAP57696.1 | putative\_secretion\_protein | BGC0000768 | Gellan\_polysaccharide\_biosynthetic\_gene\_cluster | 26 | 37.508650519 | 128 | 5.1e-29 |
| BAB78441.1 |  | BGC0000555 | Staphylococcin\_C55\_alpha\_/\_staphylococcin\_C55\_beta\_biosynthetic\_gene | 25 | 40.276816609 | 128 | 5.1e-29 |
| CAF32376.1 | putative\_ABC\_transporter,\_ATP-binding\_protein | BGC0000712 | Paromomycin\_biosynthetic\_gene\_cluster | 25 | 36.7474048443 | 128 | 8.8e-29 |
| CAJ88629.1 | putative\_netropsin\_resistance\_protein\_subunit\_2 | BGC0000327 | Congocidine\_biosynthetic\_gene\_cluster | 25 | 38.8235294118 | 128 | 8.8e-29 |
| CAB38879.1 | putative\_ABC\_transporter | BGC0000315 | Calcium-dependent\_antibiotic\_biosynthetic\_gene\_cluster | 23 | 39.8615916955 | 127 | 1.1e-28 |
| BAJ05895.1 | putative\_ABC\_multidrug\_resistance\_transporter | BGC0000872 | A-90289\_biosynthetic\_gene\_cluster | 26 | 38.1314878893 | 127 | 1.1e-28 |
| BAH43768.1 | ABC\_transporter\_ATP-binding/permease\_protein | BGC0000452 | Tyrocidine\_biosynthetic\_gene\_cluster | 26 | 38.615916955 | 127 | 1.1e-28 |
| ABV83219.1 | CppH | BGC0000116 | Nystatin-like\_Pseudonocardia\_polyene\_biosynthetic\_gene\_cluster | 25 | 38.2698961938 | 126 | 2e-28 |
| CAP20371.1 | ABC\_transporter\_ATP-binding\_membrane\_protein | BGC0000347 | Equibactin\_biosynthetic\_gene\_cluster | 25 | 35.2249134948 | 126 | 2e-28 |
| AGM16415.1 | paenibacterin\_ABC-transporter\_D | BGC0000400 | Paenibacterin\_biosynthetic\_gene\_cluster | 25 | 38.9619377163 | 126 | 3.3e-28 |
| ALV82355.1 | ABC\_transporter | BGC0001370 | Lipopeptide\_8D1-1\_/\_lipopeptide\_8D1-2\_biosynthetic\_gene\_cluster | 24 | 39.8615916955 | 126 | 3.3e-28 |
| CAD89782.1 | hypothetical\_protein | BGC0001010 | Melithiazol\_biosynthetic\_gene\_cluster | 25 | 39.1003460208 | 125 | 4.4e-28 |
| CAB60262.1 | MrsT\_protein | BGC0000527 | Mersacidin\_biosynthetic\_gene\_cluster | 23 | 40.0 | 125 | 5.7e-28 |
| WP\_009985592.1 | peptidase\_C39 | BGC0001311 | Flavecin\_biosynthetic\_gene\_cluster | 25 | 38.4775086505 | 125 | 5.7e-28 |
| AHF21240.1 | CrnT | BGC0000500 | Carnolysin\_A1\_/\_carnolysin\_A2\_biosynthetic\_gene\_cluster | 25 | 31.6262975779 | 124 | 7.4e-28 |
| AAX31531.1 | putative\_ABC\_transporter | BGC0000336 | Daptomycin\_biosynthetic\_gene\_cluster | 22 | 37.3702422145 | 124 | 9.7e-28 |
| ADC96668.1 | ABC\_transporter | BGC0001076 | Liposidomycin\_biosynthetic\_gene\_cluster | 26 | 39.5155709343 | 123 | 2.2e-27 |
| ABW74893.1 | ABC\_transporter | BGC0000759 | Diutan\_polysaccharide\_biosynthetic\_gene\_cluster | 25 | 36.955017301 | 123 | 2.8e-27 |
| ALE27499.1 | ABC\_transporter | BGC0001292 | A33853\_biosynthetic\_gene\_cluster | 27 | 23.8062283737 | 122 | 3.7e-27 |
| EDY04298.1 | ABC\_transporter-related\_protein | BGC0000516 | Geobacillin\_II\_biosynthetic\_gene\_cluster | 23 | 39.5155709343 | 122 | 4.8e-27 |
| WP\_043270371.1 | ABC\_transporter\_ATPase | BGC0001188 | Legonaridin\_biosynthetic\_gene\_cluster | 26 | 37.3702422145 | 121 | 6.3e-27 |
| BAL72550.1 | lariatin\_biosynthetic\_protein | BGC0000575 | Lariatin\_biosynthetic\_gene\_cluster | 25 | 35.7093425606 | 121 | 6.3e-27 |
| ACQ63630.1 | ABC\_multidrug\_resistance\_transporter | BGC0000875 | Caprazamycin\_biosynthetic\_gene\_cluster | 26 | 38.062283737 | 120 | 1.8e-26 |
| AAS92552.1 | SirA | BGC0001044 | Sirodesmin\_biosynthetic\_gene\_cluster | 25 | 41.1764705882 | 120 | 1.8e-26 |
| BAH43767.1 | ABC\_transporter\_ATP\_binding/permease\_protein | BGC0000452 | Tyrocidine\_biosynthetic\_gene\_cluster | 23 | 39.0311418685 | 120 | 1.8e-26 |
| BAP05577.1 | CalU | BGC0000967 | Calyculin\_biosynthetic\_gene\_cluster | 25 | 34.8788927336 | 119 | 2.4e-26 |
| AHH99931.1 | ABC\_transporter\_protein | BGC0000002 | Aculeximycin\_biosynthetic\_gene\_cluster | 24 | 35.9169550173 | 118 | 9.1e-26 |
| AHJ59536.1 | ABC\_transporter\_protein | BGC0000502 | Cerecidin\_/\_cerecidin\_A1\_/\_cerecidin\_A2\_/\_cerecidin\_A3\_/\_cerecidin | 23 | 37.4394463668 | 118 | 9.1e-26 |
| ABS50189.1 | putative\_ABC\_transporter\_permease/ATP-binding\_protein | BGC0000410 | Pseudomonine\_biosynthetic\_gene\_cluster | 26 | 34.8788927336 | 116 | 2e-25 |
| AJM89737.1 | PmxD | BGC0001192 | Colistin\_biosynthetic\_gene\_cluster | 26 | 35.0173010381 | 116 | 3.4e-25 |
| BAE56595.1 |  | BGC0001123 | Aspirochlorine\_biosynthetic\_gene\_cluster | 23 | 37.508650519 | 116 | 3.4e-25 |
| ACA97579.1 | PmxD | BGC0000408 | Polymyxin\_biosynthetic\_gene\_cluster | 26 | 35.0173010381 | 115 | 4.5e-25 |
| CAJ87587.1 | putative\_ABC\_transporter\_protein | BGC0001055 | Yersiniabactin\_biosynthetic\_gene\_cluster | 26 | 24.6366782007 | 115 | 5.9e-25 |
| AEZ51519.1 | PmxD | BGC0001153 | Polymyxin\_biosynthetic\_gene\_cluster | 26 | 35.0173010381 | 114 | 7.7e-25 |
| CAA11793.1 | PCZA363.2 | BGC0000322 | Chloroeremomycin\_biosynthetic\_gene\_cluster | 24 | 40.0692041522 | 114 | 7.7e-25 |
| AGS77319.1 | ABC\_transporter | BGC0001178 | UK-68,597\_biosynthetic\_gene\_cluster | 24 | 38.892733564 | 114 | 1e-24 |
| AAK73499.1 | AmphH | BGC0000015 | Amphotericin\_biosynthetic\_gene\_cluster | 25 | 38.339100346 | 114 | 1.3e-24 |
| AAF71764.1 | NysH | BGC0000115 | Nystatin\_biosynthetic\_gene\_cluster | 26 | 38.5467128028 | 114 | 1.3e-24 |
| CAG15016.1 | ABC\_transporter | BGC0000441 | Teicoplanin\_biosynthetic\_gene\_cluster | 23 | 39.1003460208 | 113 | 1.7e-24 |
| CAE53357.1 | ABC\_transporter | BGC0000440 | Teicoplanin\_biosynthetic\_gene\_cluster | 23 | 39.1003460208 | 113 | 1.7e-24 |
| CAC17507.1 | putative\_ABC\_transporter\_ATP-binding\_protein | BGC0000324 | Coelibactin\_biosynthetic\_gene\_cluster | 27 | 37.9238754325 | 113 | 2.2e-24 |
| AAM80540.1 | StaU | BGC0000290 | A47934\_biosynthetic\_gene\_cluster | 23 | 38.9619377163 | 113 | 2.9e-24 |
| CAD15511.1 | probable\_composite\_atp-binding\_transmembrane\_abc\_transporter\_protein | BGC0001014 | Micacocidin\_biosynthetic\_gene\_cluster | 26 | 34.8096885813 | 112 | 3.8e-24 |
| CAG25756.1 | putative\_ABC\_transporter\_ATP-binding\_protein | BGC0000311 | Balhimycin\_biosynthetic\_gene\_cluster | 23 | 39.9307958478 | 112 | 3.8e-24 |
| AIG79244.1 | Conserved\_putative\_membrane\_protein | BGC0000419 | Ristomycin\_A\_biosynthetic\_gene\_cluster | 23 | 39.169550173 | 111 | 8.5e-24 |
| AIS24846.1 | Dst20 | BGC0001147 | Distamycin\_biosynthetic\_gene\_cluster | 28 | 31.7647058824 | 110 | 1.4e-23 |
| AIE77056.1 | ABC-transporter | BGC0000418 | Ristocetin\_biosynthetic\_gene\_cluster | 24 | 39.3771626298 | 109 | 3.2e-23 |
| AFJ14796.1 | PlpG | BGC0000403 | Pelgipeptin\_biosynthetic\_gene\_cluster | 24 | 37.8546712803 | 109 | 4.2e-23 |
| AEI58864.1 | ABC\_transporter | BGC0000455 | Vancomycin\_biosynthetic\_gene\_cluster | 23 | 41.7993079585 | 108 | 5.5e-23 |
| AAK27372.1 | ABC\_transporter | BGC0000623 | Plantaricin\_W\_alpha\_/\_plantaricin\_W\_beta\_biosynthetic\_gene\_cluster | 24 | 32.1799307958 | 108 | 5.5e-23 |
| CAA12529.1 | ExsA\_protein | BGC0000802 | Succinoglycan\_biosynthetic\_gene\_cluster | 25 | 32.0415224913 | 108 | 7.2e-23 |
| AGL93176.1 | ABC\_transporter\_ATP-binding\_protein | BGC0000560 | Subtilomycin\_biosynthetic\_gene\_cluster | 23 | 34.8788927336 | 107 | 1.2e-22 |
| AHZ61877.1 | Lom43 | BGC0000240 | Lomaiviticin\_biosynthetic\_gene\_cluster | 23 | 38.2698961938 | 107 | 1.2e-22 |
| AJM89736.1 | PmxC | BGC0001192 | Colistin\_biosynthetic\_gene\_cluster | 24 | 37.23183391 | 106 | 2.7e-22 |
| CAJ88628.1 | putative\_netropsin\_resistance\_protein\_subunit\_1 | BGC0000327 | Congocidine\_biosynthetic\_gene\_cluster | 24 | 36.678200692 | 106 | 3.6e-22 |
| ABS50188.1 | putative\_ABC\_transporter\_permease/ATP-binding\_protein | BGC0000410 | Pseudomonine\_biosynthetic\_gene\_cluster | 25 | 38.2006920415 | 105 | 6.1e-22 |
| AEZ51518.1 | PmxC | BGC0001153 | Polymyxin\_biosynthetic\_gene\_cluster | 24 | 38.4775086505 | 104 | 8e-22 |
| ABP54659.1 | ABC\_transporter\_related | BGC0000241 | Lomaiviticin\_biosynthetic\_gene\_cluster | 24 | 38.062283737 | 102 | 3.9e-21 |
| BAE07080.1 | possible\_ABC\_transporter | BGC0000693 | Butirosin\_biosynthetic\_gene\_cluster | 27 | 25.8823529412 | 100 | 2e-20 |
| CCC55904.1 | putative\_ABC\_transporter,\_ATPase\_and\_permease\_component | BGC0000973 | Collismycin\_A\_biosynthetic\_gene\_cluster | 23 | 36.0553633218 | 99 | 4.4e-20 |
| CAA11515.1 | colicin\_V\_exporter\_protein | BGC0000584 | Colicin\_V\_biosynthetic\_gene\_cluster | 27 | 20.3460207612 | 99 | 4.4e-20 |
| AAA62651.1 |  | BGC0000504 | Cytolysin\_ClyLl\_/\_cytolysin\_ClyLs\_biosynthetic\_gene\_cluster | 24 | 39.6539792388 | 97 | 1.7e-19 |
| AEH59056.1 | YvcC/ABC\_transporter | BGC0000385 | Lysobactin\_biosynthetic\_gene\_cluster | 23 | 39.2387543253 | 95 | 4.8e-19 |
| AAG32538.1 | SalT | BGC0000548 | Salivaricin\_A\_biosynthetic\_gene\_cluster | 22 | 39.9307958478 | 92 | 3.1e-18 |
| ACP43452.1 | McnB | BGC0000590 | Microcin\_N\_biosynthetic\_gene\_cluster | 24 | 33.9792387543 | 91 | 7e-18 |
| AFS60105.1 | PaenT | BGC0000540 | Paenibacillin\_biosynthetic\_gene\_cluster | 23 | 37.508650519 | 91 | 1.2e-17 |
| CAE55703.1 | probable\_microcin\_H47\_secretion\_ATP-binding\_protein\_mchF | BGC0000589 | Microcin\_M\_biosynthetic\_gene\_cluster | 23 | 35.5709342561 | 89 | 2.6e-17 |
| AAC63532.1 | sublancin\_transporter\_protein | BGC0000558 | Sublancin\_168\_biosynthetic\_gene\_cluster | 21 | 36.4705882353 | 89 | 2.6e-17 |
| CAJ44959.1 | MchF\_protein | BGC0000587 | Microcin\_H47\_biosynthetic\_gene\_cluster | 23 | 35.5709342561 | 89 | 4.5e-17 |
| CBW74823.1 | Multidrug\_resistance\_ABC\_transporter\_ATP-binding\_and\_permease\_protein | BGC0000571 | Burhizin\_biosynthetic\_gene\_cluster | 28 | 20.1384083045 | 88 | 5.9e-17 |
| CAC17506.1 | putative\_ABC\_transporter\_ATP-binding\_protein | BGC0000324 | Coelibactin\_biosynthetic\_gene\_cluster | 23 | 35.9169550173 | 88 | 5.9e-17 |
| CAG38699.1 | putative\_ABC-transporter,\_ATP-binding\_protein | BGC0000708 | Lividomycin\_biosynthetic\_gene\_cluster | 23 | 27.9584775087 | 86 | 2.2e-16 |
| BAC87910.1 | probable\_ABC\_transporter | BGC0000294 | Acinetobactin\_biosynthetic\_gene\_cluster | 24 | 35.0173010381 | 86 | 2.9e-16 |
| AJF34465.1 | ABC\_transporter | BGC0001207 | Teixobactin\_biosynthetic\_gene\_cluster | 23 | 34.9480968858 | 86 | 3.8e-16 |
| CAI47638.1 | putative\_ABC-type\_aminoglycoside\_exporter | BGC0000692 | Apramycin\_biosynthetic\_gene\_cluster | 23 | 29.5501730104 | 85 | 5e-16 |
| CAP20370.1 | ABC\_transporter,\_ATP-binding\_membrane\_protein | BGC0000347 | Equibactin\_biosynthetic\_gene\_cluster | 24 | 31.9031141869 | 85 | 5e-16 |
| CAX48974.1 | ABC-transporter | BGC0000519 | Labyrinthopeptin\_A1,A3\_/\_labyrinthopeptin\_A2\_biosynthetic\_gene | 25 | 36.3321799308 | 84 | 8.5e-16 |
| AFD30963.1 | CrmT2 | BGC0000966 | Caerulomycin\_A\_biosynthetic\_gene\_cluster | 23 | 35.5709342561 | 84 | 8.5e-16 |
| AAL08400.1 | MceG | BGC0000586 | Microcin\_E492\_biosynthetic\_gene\_cluster | 22 | 33.0795847751 | 84 | 1.5e-15 |
| AAP03990.1 | putative\_microcin\_L\_transport\_protein | BGC0000588 | Microcin\_L\_biosynthetic\_gene\_cluster | 22 | 35.5709342561 | 83 | 2.5e-15 |
| AFW17836.1 | PaeT | BGC0000541 | Paenicidin\_A\_biosynthetic\_gene\_cluster | 23 | 39.1003460208 | 82 | 4.2e-15 |
| CAF31838.1 | putative\_hygromycin\_B\_exporter | BGC0000699 | Hygromycin\_B\_biosynthetic\_gene\_cluster | 25 | 31.2110726644 | 81 | 7.2e-15 |
| CAD91219.1 | putative\_ABC\_transporter,\_Dbv24 | BGC0000289 | A40926\_biosynthetic\_gene\_cluster | 25 | 24.4290657439 | 76 | 2.3e-13 |
| ACY56719.1 | ThnT | BGC0000600 | Thurincin\_H\_biosynthetic\_gene\_cluster | 21 | 33.9100346021 | 76 | 4e-13 |
| ACA97578.1 | PmxC | BGC0000408 | Polymyxin\_biosynthetic\_gene\_cluster | 23 | 28.7889273356 | 76 | 4e-13 |
| ctg1\_orf5 |  | BGC0000321 | Chaetocin\_biosynthetic\_gene\_cluster | 22 | 64.4982698962 | 75 | 5.2e-13 |
| AHW81482.1 | hypothetical\_protein | BGC0000934 | Endophenazines\_biosynthetic\_gene\_cluster | 25 | 27.9584775087 | 74 | 8.8e-13 |
| AAG28766.1 | CoaD | BGC0000617 | Coagulin\_biosynthetic\_gene\_cluster | 24 | 30.2422145329 | 70 | 2.2e-11 |
| ALX37944.1 | GaeT | BGC0001388 | Gassericin\_E\_biosynthetic\_gene\_cluster | 21 | 26.0899653979 | 69 | 3.7e-11 |
| BAM09402.1 | putative\_ABC-transporter | BGC0000619 | Gassericin\_T\_biosynthetic\_gene\_cluster | 21 | 26.0899653979 | 69 | 4.8e-11 |
| EMC15128.1 | ABC\_transporter,\_ATP-binding\_and\_permease\_protein | BGC0000530 | Mutacin\_B-Ny266\_biosynthetic\_gene\_cluster | 25 | 20.4152249135 | 65 | 5.3e-10 |
| ACM68694.1 | AerN | BGC0000298 | Aeruginosin\_biosynthetic\_gene\_cluster | 23 | 22.1453287197 | 62 | 4.5e-09 |
| CAM03501.1 | putative\_ABC\_transporter | BGC0000513 | Erythreapeptin\_biosynthetic\_gene\_cluster | 22 | 27.8200692042 | 56 | 2.5e-07 |
